# Supplementary material for: Eighty-eight variants highlight the role of T cell regulation and airway remodeling in asthma pathogenesis
Source: Nat Commun. 2020 Jan 20;11:393. doi: 10.1038/s41467-019-14144-8 (PMC6971247; doi:10.1038/s41467-019-14144-8)
Supplement: Supplementary file 1 — Supplementary Information [file 41467_2019_14144_MOESM1_ESM.pdf]

Supplementary information

Eighty-eight variants highlight the role of T cell regulation and airway remodeling in asthma pathogenesis

Thorunn A Olafsdottir[1,2], Fannar Theodors[1], Kristbjorg Bjarnadottir[1], Unnur Steina Bjornsdottir[3,4], Arna B Agustsdottir[1], Olafur A Stefansson[1], Erna V Ivarsdottir[1,5], Jon K Sigurdsson[1], Stefania Benonisdottir[1], Gudmundur I Eyjolfsson[6], David Gislason[4,7], Thorarinn Gislason[2,8], Steinunn Guðmundsdóttir[1], Arnaldur Gylfason[1], Bjarni V Halldorsson[1,9], Gisli H Halldorsson[1], Thorhildur Juliusdottir[1], Anna M Kristinsdottir[1], Dora Ludviksdottir[2,7], Bjorn R Ludviksson[2,10], Gisli Masson[1], Kristjan Norland[1], Pall T Onundarson[2,11], Isleifur Olafsson[12], Olof Sigurdardottir[2,13], Lilja Stefansdottir[1], Gardar Sveinbjornsson[1], Vinicius Tragante[1,14], Daniel F Gudbjartsson[1,5], Gudmar Thorleifsson[1], Patrick Sulem[1], Unnur Thorsteinsdottir[1,2], Gudmundur L Norddahl[1], Ingileif Jonsdottir[1,2] and Kari Stefansson[1,2]

1. deCODE genetics/Amgen, Inc., Reykjavik, Iceland
2. Faculty of Medicine, School of Health Sciences, University of Iceland, Reykjavik, Iceland
3. Department of Medicine, Landspitali, The National University Hospital of Iceland, Reykjavik, Iceland
4. The Medical Center Mjodd, Reykjavik, Iceland
5. School of Engineering and Natural Sciences, University of Iceland, Reykjavik, Iceland
6. The Laboratory in Mjodd, RAM, Reykjavik, Iceland
7. Department of Respiratory Medicine and Sleep, Landspitali, The National University Hospital of Iceland, Reykjavik, Iceland
8. Department of Sleep, Landspitali, The National University Hospital of Iceland, Reykjavik, Iceland
9. School of Science and Engineering, Reykjavik University, Reykjavík, Iceland
10. Department of Immunology, Landspitali, The National University Hospital of Iceland, Reykjavik, Iceland
11. Department of Laboratory Hematology, Landspitali, The National University Hospital of Iceland, Reykjavik, Iceland
12. Department of Clinical Biochemistry, Landspitali, The National University Hospital of Iceland, Reykjavik, Iceland
13. Department of Clinical Biochemistry, Akureyri Hospital, Akureyri, Iceland
14. Department of Cardiology, Division Heart & Lungs, University Medical Center Utrecht, University of Utrecht, Utrecht, The Netherlands.

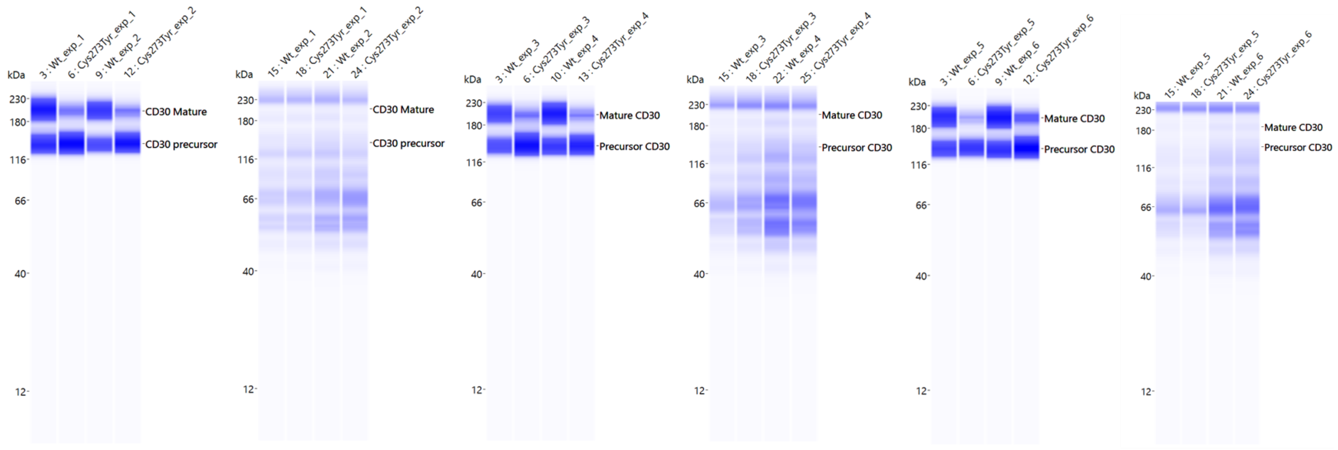

Suplementary figure 1: CD30 expression in HeLa cells overexpressing wild-type or variant p.Cys273Tyr CD30.

Protein simple WES analysis of CD30 expression in cell lysate from HeLa cells overexpressing wild-type or variant p.Cys273Tyr CD30. One representative lane is shown for each triplicate of lysate run and corresponding total protein analysis of those samples. CD30 and total protein quantifications were run in separate wells from the same lysis preparation (N=6).

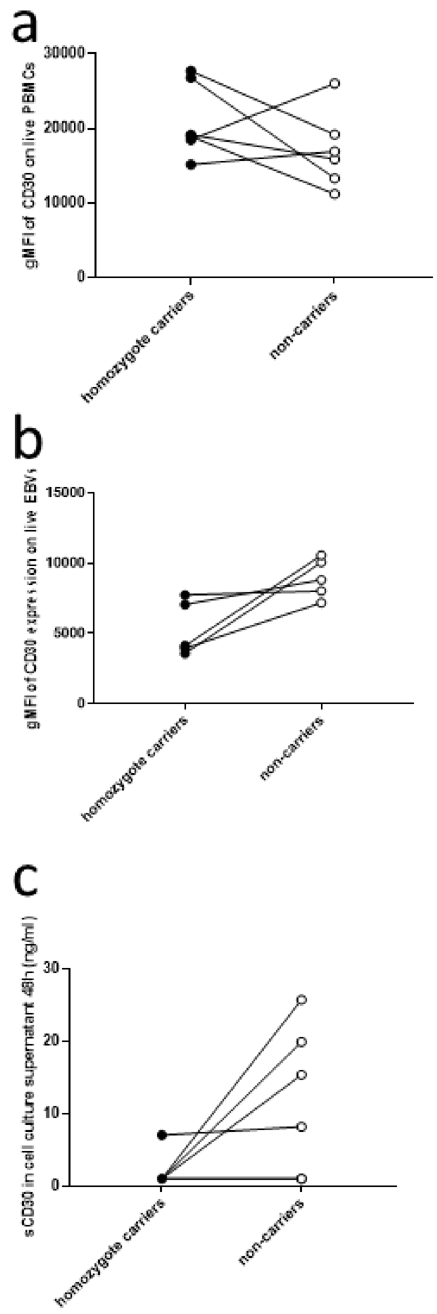

Supplementary figure 2: Surface expression of CD30 on PBMC and EBV transformed lymphoblasts from homozygous p.Cys273Tyr carriers age and gender matched non-carriers.

(**a**) Surface expression of CD30 on stimulated PBMCs from homozygous carriers of p.Cys273Tyr and age and gender matched non-carriers measured by flow cytometry as gMFI. (**b**) Surface expression of CD30 on EBV transformed lymphoblasts from a homozygous carriers and a non-carrier of p.Cys273Tyr. (**c**) Soluble CD30 (sCD30) levels (ng/ml) in activated PBMC culture supernatant from homoozygotes and non-carriers of p.Cys273Tyr.

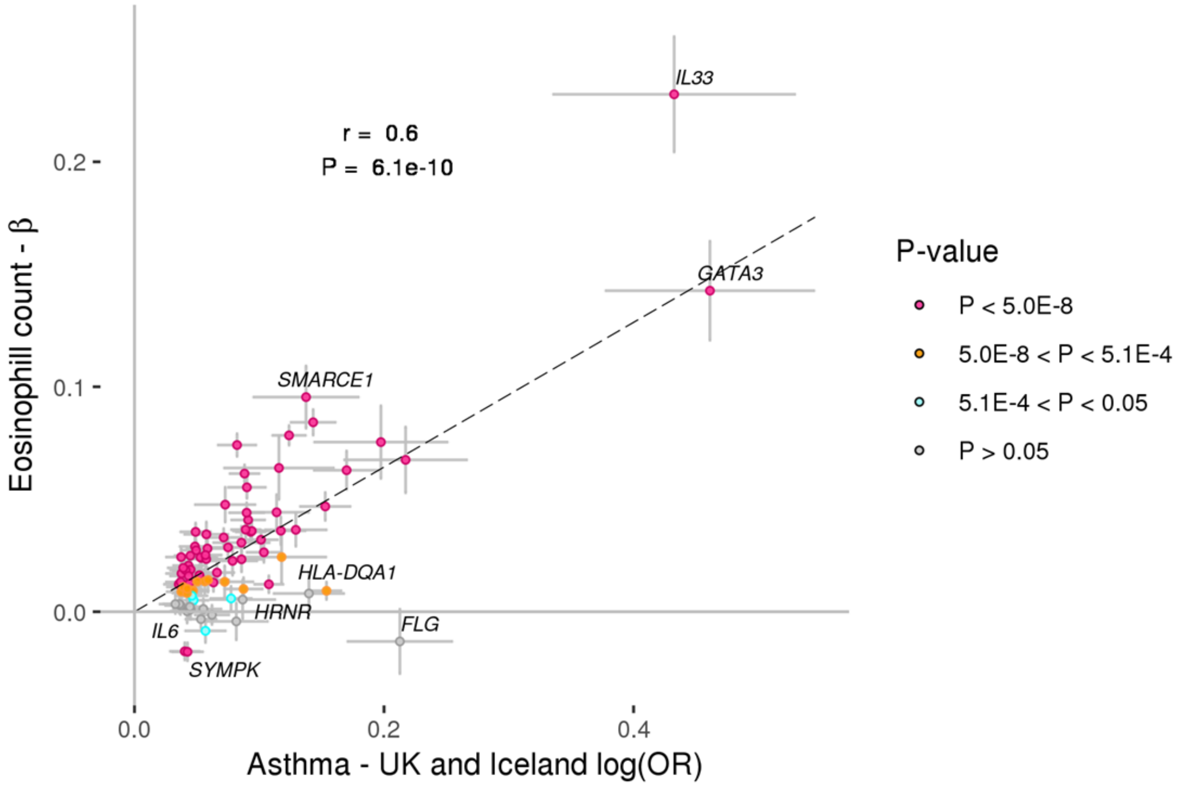

Suplementary figure 3: Effects of the 88 asthma variants on asthma and eosinophil count. The x-axis of the scatter plot shows the logarithm of the asthma odds ratios found in Icelandic and UK meta-analysis and the y-axis shows the age adjusted effect (in SD) on Eosinophil count in a combined data set of Iceland and UK. All effects are shown for the asthma risk increasing allele. Error bars represent 95% confidence intervals. The line represents results from a simple linear regression through the origin using MAF (1-MAF) as weights and the weighted correlation coefficient (r) and *P*-value (t-test) are shown in the graph.

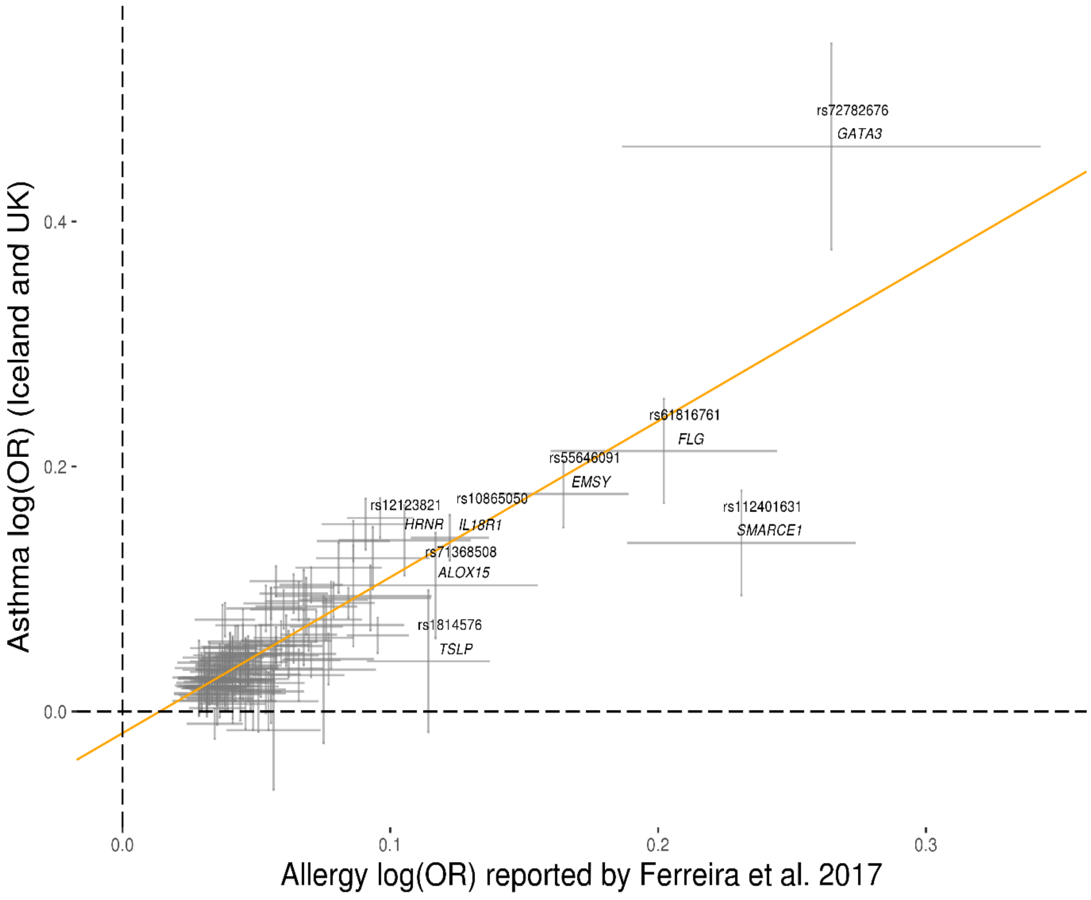

Supplementary figue 4: Effects of reported allergy variants (Ferreira et al. 2017) on asthma and allergy. Scatter plot showing 134 previously reported allergy SNPs. The x-axis shows the logarithm of their allergy odds ratios reported by Ferreira et al. 2017 and the y-axis shows the logarithm of estimated odds ratios for asthma in a combined data set of Iceland and UK (Cases: $N_{Iceland}$=16,247; N_{UK}=52,972). All effects are shown for the allergy risk increasing allele. Variants associated with the largest allergy risk are labeled. Error bars represent 95% confidence intervals. The orange solid line, y = -0.02+ 1.27x, represents results from a simple linear regression using MAF(1-MAF) as weights with R = 0.77 (P = 6.0×10-28; t-test).

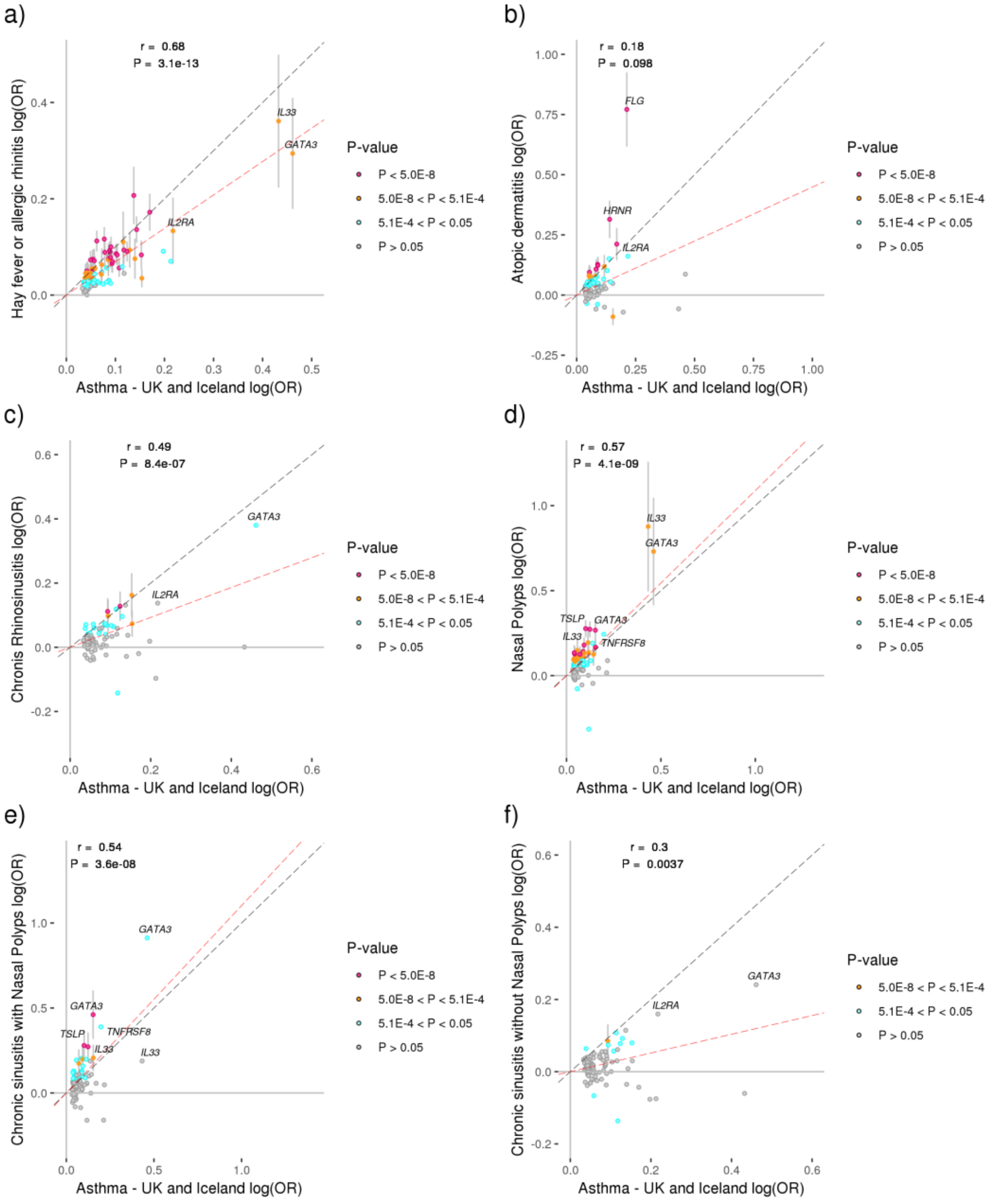

Supplementary Figure 5. Effects of the 89 asthma variants on asthma and various allergic phenotypes. The x-axis of the scatter plot shows the logarithm of the asthma odds ratios found in Icelandic and UK meta-analysis and the y-axis shows the logarithm of estimated odds ratios for (**a**) hay fever or allergic rhinitis, (**b**) Atopic dermatitis, (**c**) Chronic rhinosinusitis, (**d**) Nasal Polyps, (**e**) Chronic rhinosinusitis with Nasal Polyps and (**f**) Chronic rhinosinusitis with Nasal Polyps in a combined data set of Iceland and UK. Effects of all allergic phenotypes are based on Icelandic and UK meta-analysis except for Atopic dermatitis that is based on Icelandic and external data meta-analysis (Paternoster et. al, 2015) as described in materials and methods. All effects are shown for the asthma risk increasing allele. Error bars represent 95% confidence intervals. The line represents results from a simple linear regression through the origin using MAF (1-MAF) as weights and the weighted correlation coefficient (r) and *P*-value (t-test) are shown in the graphs.

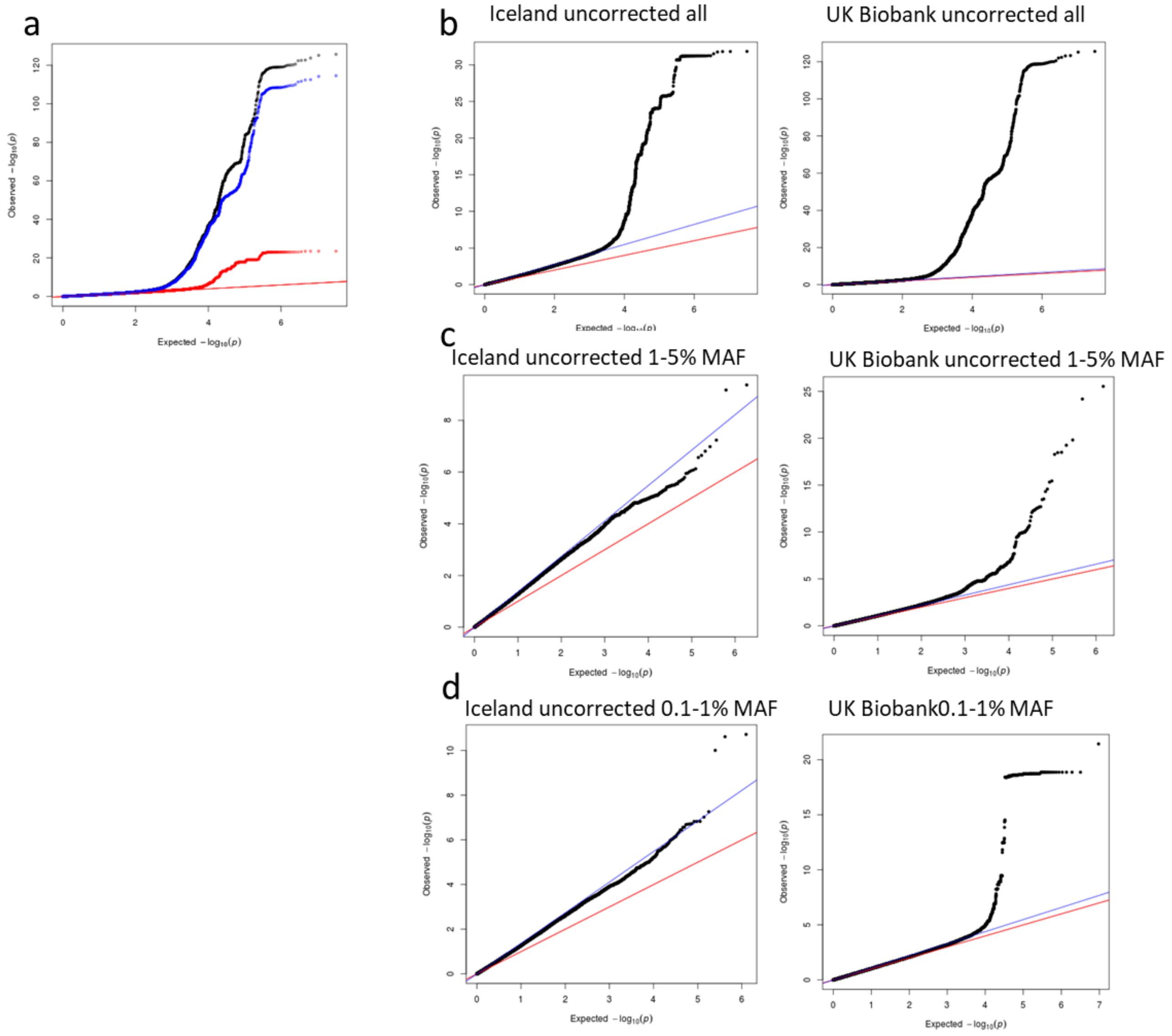

Supplementary Figure 6. Quantile-quantile plot (QQ-plot) showing chi-square statistics for (a) all variants corrected for genomic control, (b) all variants not corrected for genomic control, (c) low frequency variants (1%-5%) not corrected for genomic control and (d) rare variants (0.1%-1%) not corrected for genomic control. The different colors in (a) representvariants found in Iceland and UK cohorts for the GWAS of Asthma in Iceland (red) the UK (blue) and meta-analysis of the two cohorts (black). The red diagonal line represents expected distribution assuming no inflation of the chi-square statistics. The blue line in figures b-d represent the genomic correction factor; 1.097 for UK Biobank and 1.370 for Iceland

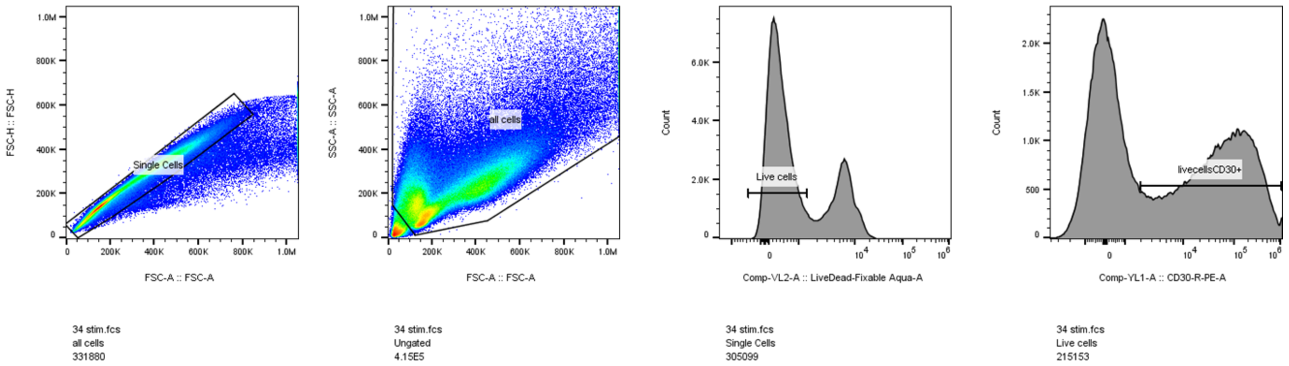

Supplementary figure 7: Gating strategy used for facs analysis to determine geometric mean fluorescence intensity of CD30 positive live cells presented in Fig. 2c,e and Supplementary Fig. 2a,b.

Supplementary table 1. Correlation of the effect of asthma associating variants in other asthma/allergy phenotypes. All the correlation is performed on meta-analysis of Icelandic and UK data (number of participants in each country for the different phenotypes is shown in the table).
The table depicts the weighted correlation between effects of 88 indpendent asthma variants and the 11 phenotypes shown in the first column. MAF(1-MAF) were used as weights when computing the correlation.

Phenotype	P	wCorr	Iceland (n)	UK (n)	External****
Asthma ICD10 codes diagnosis+	5.3E-81	0.97	13190	26152	-
Asthma self-reported+	3.30E-120	0.99	6281	47882	-
Early-Onset Asthma*	1.4E0-27	0.86	4394	12723	-
Late-Onset Asthma**	1.1E-22	0.82	12329	29731	-
Allergic Asthma	8.4E-19	0.77	3572	542	-
Hay fever or allergic rhinitis***	3.1E-13	0.68	7460	24804	-
Nasal Polyps	4.10E-09	0.58	1874	3191	-
Chronic sinusitis with Nasal Polyps	3.6E-08	0.54	906	672	-
Chronis Rhinosinusitis	8.4E-07	0.49	3886	2420	-
Chronic sinusitis without Nasal Polyps	3.70E-03	0.3	3061	1748	-
Atopic dermatitis	0.32	0.11	8325	-	21399

+There is an overlap between the ICD10 codes diagnosis and Astma self reported lists both in Iceland (N=3224) and in the UK (N=21092)
*Early onset is defined as the first diagnosis < 18 years of age
**Late onset is defined as the first diagnosis ≥ 18 years of age. Late onset asthma diagnosis from Iceland contains both where the first diagnosis was at or after 18 years of age and where the first diagnosis is unknown
*Hayfever or allergic rhinitis combines doctoral diagnosis of allergic rhinitis from Iceland and a questionnare data from UK Biobank on hayfever or allergic rhinitis (Non-cancer-illness code 1387)
****Due the few ICD10 code (L20) defined Atopic dermatitis cases reported in UKB, the AD association is based on cases defined in Iceland (N=8,325) and the largest GWAS on AD to date (N=21,399; PMID:26482879)

Supplementary Table 2. Genetic correlation calculated between asthma and allergic phenotypes in Iceland and UK biobank

Phenotype (N Iceland)	Phenotype (N UKB)	rg	se	P
Asthma (16,247)	Asthma (52,942)	0.8899	0.097	4.60E-20
Hay fever or allergic rhinitis (7,460)	Hay fever or allergic rhinitis (24,804)	0.9874	0.1266	6.30E-15
Nasal Polyps (1,874)	Nasal Polyps (3,191)	1.0663	0.3301	0.0012
Chronic sinusitis with Nasal Polyps (906)	Chronic sinusitis with Nasal Polyps (672)	0.4068	0.3035	0.1801
Chronis Rhinosinusitis (3,886)	Chronis Rhinosinusitis (2,420)	1.0763	0.6307	0.0879
Chronic sinusitis without Nasal Polyps (3,061)	Chronic sinusitis without Nasal Polyps (1,748)*	-	-	-
Atopic dermatitis (8,325)	Atopic dermatitis (external)	0.9135	0.2522	0.0003
Asthma (16,247)	Hay fever or allergic rhinitis (24,804)	0.4244	0.0936	5.83E-06
Asthma (16,247)	Nasal Polyps (3,191)	0.5442	0.1286	2.32E-05
Asthma (16,247)	Chronic sinusitis with Nasal Polyps (672)	0.3053	0.172	0.076
Asthma (16,247)	Chronis Rhinosinusitis (2,420)	0.3802	0.2545	1.35E-01
Asthma (16,247)	Chronic sinusitis without Nasal Polyps (1,748)*	-	-	-
Asthma (16,247)	Atopic dermatitis (external; N=21,399)	0.3488	0.1333	0.0089
Hay fever or allergic rhinitis (7,460)	Asthma (52,942)	0.7985	0.1033	1.07E-14
Nasal Polyps (1,874)	Asthma (52,942)	0.4312	0.1414	0.0023
Chronic sinusitis with Nasal Polyps (906)	Asthma (52,942)	0.5248	0.193	0.0065
Chronis Rhinosinusitis (3,886)	Asthma (52,942)	0.4733	0.196	0.016
Chronic sinusitis without Nasal Polyps (3,061)	Asthma (52,942)	0.2662	0.1375	0.0529
Atopic dermatitis (8,325)	Asthma (52,942)	0.5617	0.1401	6,07E-05
Atopic dermatitis (external; N=21,399)	Asthma (52,942)	0.4576	0.0671	9,38E-12

* Chronic Sinusitis without Nasal polyps list from the UKB did not yield any results above noise level.

Supplementary table 3: Characteristics of Icelandic and UKB asthma cohorts.

Cohorts	N	Yob (SD)	Male N (%)
Iceland cases	16,247	1965.4 (26.5)	6604 (40.6)
Iceland controls	346,486	1968.7 (29.0)	176,981 (51.1)
UK cases	52,942	1951.6 (8.2)	22,520 (42.5)
UK controls	355,713	1951.1 (8.0)	165,199 (46.4)

In the UK individuals aged between 40 and 69 were recruited whereas there was no age restriction in the Icelandic recruitment.

Supplementary table 4: List of primers used in the study

**Primer**	**Sequence**
CD30_C273T_F	5‘AAGACGCCATATGCATGGAAC‘3
CD30_C273T_R	5‘CTCCACAAGGTCATCTCG‘3
