# Supplementary material for: Eighty-eight variants highlight the role of T cell regulation and airway remodeling in asthma pathogenesis
Source: Nat Commun. 2020 Jan 20;11:393. doi: 10.1038/s41467-019-14144-8 (PMC6971247; doi:10.1038/s41467-019-14144-8)
Supplement: Supplementary file 2 — Reporting Summary [file 41467_2019_14144_MOESM2_ESM.pdf]

nature research

Corresponding author(s): NCOMMS-18-38522-D

Last updated by author(s): 15/11/19

Reporting Summary

Nature Research wishes to improve the reproducibility of the work that we publish. This form provides structure for consistency and transparency in reporting. For further information on Nature Research policies, see Authors & Referees and the Editorial Policy Checklist.

Statistics

For all statistical analyses, confirm that the following items are present in the figure legend, table legend, main text, or Methods section.

n/a	Confirmed	
☐	☒	The exact sample size (*n*) for each experimental group/condition, given as a discrete number and unit of measurement
☐	☒	A statement on whether measurements were taken from distinct samples or whether the same sample was measured repeatedly
☐	☒	The statistical test(s) used AND whether they are one- or two-sided<br>*Only common tests should be described solely by name; describe more complex techniques in the Methods section.*
☐	☒	A description of all covariates tested
☐	☒	A description of any assumptions or corrections, such as tests of normality and adjustment for multiple comparisons
☐	☒	A full description of the statistical parameters including central tendency (e.g. means) or other basic estimates (e.g. regression coefficient) AND variation (e.g. standard deviation) or associated estimates of uncertainty (e.g. confidence intervals)
☐	☒	For null hypothesis testing, the test statistic (e.g. *F*, *t*, *r*) with confidence intervals, effect sizes, degrees of freedom and *P* value noted<br>*Give P values as exact values whenever suitable.*
☒	☐	For Bayesian analysis, information on the choice of priors and Markov chain Monte Carlo settings
☐	☒	For hierarchical and complex designs, identification of the appropriate level for tests and full reporting of outcomes
☐	☒	Estimates of effect sizes (e.g. Cohen's *d*, Pearson's *r*), indicating how they were calculated

Our web collection on statistics for biologists contains articles on many of the points above.

Software and code

Policy information about availability of computer code

Data collection	The "Whole-genome sequencing and imputation" subsection in materials and methods section contains the following information:<br>"The GWASs in Iceland were performed with 32.5 million markers identified through whole-genome sequencing of 15,520 Icelanders to an average genome-wide coverage of 34X and subsequently imputed into 151,677 chip-typed individuals, as well as their first and second degree relatives. The imputation has been extensively described in recent publications45. Genotyping of UKB samples was performed using a custom-made Affymetrix chip, UK BiLEVE Axiom46, and with the Affymetrix UK Biobank Axiom array47. Imputation was performed by the Wellcome Trust Centre for Human Genetics, using the Haplotype Reference Consortium (HRC) and the UK10K haplotype resources. This yields a total of 96 million imputed variants, however only 27 million variants imputed using the HRC reference set passed the quality filters used in our study."
Data analysis	The "Code availability" subsection in materials and methods section contains the following information:<br>"We used publicly available software (URLs listed below) in conjunction with the above described algorithms in the sequencing processing pipeline (Whole-genome sequencing, Association testing, RNA-seq mapping and analysis):<br>BWA 0.7.10 mem, https://github.com/lh3/bwa 396<br>GenomeAnalysisTKLite 2.3.9, https://github.com/broadgsa/gatk/ 397<br>Picard tools 1.117, https://broadinstitute.github.io/picard/ 398<br>SAMtools 1.3, http://samtools.github.io/ 399<br>Bedtools v2.25.0-76-g5e7c696z, https://github.com/arq5x/bedtools2/ 400<br>Variant Effect Predictor https://github.com/Ensembl/ensembl-vep 401<br>BOLT-LMM https://data.broadinstitute.org/alkesgroup/BOLT-LMM/downloads/"<br><br>Further, R was extensively used to analyze data and create plots.

For manuscripts utilizing custom algorithms or software that are central to the research but not yet described in published literature, software must be made available to editors/reviewers. We strongly encourage code deposition in a community repository (e.g. GitHub). See the Nature Research guidelines for submitting code & software for further information.

Data

Policy information about availability of data

All manuscripts must include a data availability statement. This statement should provide the following information, where applicable:
- Accession codes, unique identifiers, or web links for publicly available datasets
- A list of figures that have associated raw data
- A description of any restrictions on data availability

Sequence variants passing GATK filters have been deposited in the European Variation Archive, accession number PRJEB15197 (https://www.ebi.ac.uk/ena/data/view/PRJEB15197

Field-specific reporting

Please select the one below that is the best fit for your research. If you are not sure, read the appropriate sections before making your selection.

☒ Life sciences ☐ Behavioural & social sciences ☐ Ecological, evolutionary & environmental sciences

For a reference copy of the document with all sections, see nature.com/documents/nr-reporting-summary-flat.pdf

Life sciences study design

All studies must disclose on these points even when the disclosure is negative.

Sample size	The sample size corresponds to all available data from Iceland and UK biobank
Data exclusions	No data was excluded from the analyzes
Replication	We performed GWAS studies in two independent populations and combined the results. Results are presented for the populations independently and combined and heterogeneity of effects between gopus are assessed.
Randomization	No randomizations was used.
Blinding	Not relevant for this study, as this is a case-control GWAS study

Reporting for specific materials, systems and methods

We require information from authors about some types of materials, experimental systems and methods used in many studies. Here, indicate whether each material, system or method listed is relevant to your study. If you are not sure if a list item applies to your research, read the appropriate section before selecting a response.

Materials & experimental systems

n/a	Involved in the study	
☐	☒	Antibodies
☐	☒	Eukaryotic cell lines
☒	☐	Palaeontology
☒	☐	Animals and other organisms
☐	☒	Human research participants
☒	☐	Clinical data

Methods

n/a	Involved in the study	
☒	☐	ChIP-seq
☐	☒	Flow cytometry
☒	☐	MRI-based neuroimaging

Antibodies

Antibodies used	TNFRSF8/CD30 (E1A6Y) Rabbit mAb. Cell signaling Cat: 95620 Lot:1<br>Biolegend 333918, Biolegend 333906, Biolegend 300318, Biolegend 300530, Biolegend 353210, Biolegend 304112
Validation	Monoclonal antibody is produced by immunizing animals with a synthetic peptide corresponding to residues surrounding Leu500 of human TNFRSF8/CD30 protein. TNFRSF8/CD30 (E1A6Y) Rabbit mAb recognizes endogenous levels of total TNFRSF8/CD30 protein. Validated for Western blot by manufacturer by Western blot analysis of extracts from 293T cells, mock transfected (-) or transfected with a construct expressing Myc/DDK-tagged full-length human TNFRSF8/CD30 protein (hCD30-Myc/DDK; +), using TNFRSF8/CD30 (E1A6Y) Rabbit antibody. See certificate of analysis here:<br>https://media.cellsignal.com/coa/95620/1/95620-lot-1-coa.pdf<br>Each lot of the Biolegend antibodies used is quality control tested by immunofluorescent staining with flow cytometric analysis as by the manufacturers as stated on their website.

Eukaryotic cell lines

Policy information about cell lines

Cell line source(s)	Hela (Human cervix epetheloid carcinoma). HeLa (ECACC 93021013) Lot: 14A021
Authentication	Cells were authenticated by ECACC. See certificate of analysis here:<br>https://www.phe-culturecollections.org.uk/CofA.do?mode=view&collection=ecacc_gc&refId=93021013&formatId=1&lotNo=14A021&searchRef=9hrr6k318t4pd
Mycoplasma contamination	Cells tested negative for mycoplasma.
Commonly misidentified lines (See ICLAC register)	*Name any commonly misidentified cell lines used in the study and provide a rationale for their use.*

Human research participants

Policy information about studies involving human research participants

Population characteristics	The Icelandic data is based on whole-genome sequence data from the whole blood of 15,220 Icelanders participating in various disease projects at deCODE genetics. In addition, 151,677 Icelanders have been genotyped using Illumina SNP chips and genotype probabillities for untyped relatives have been calculated based on Icelandic genealogy. The UK data is from the UK Biobank, a large prospective cohort study of aprox. 500,000 individuals from across the United Kingdom, aged between 40-69 at recruitment. As only 42 individuals had the ICD10 code for Atopic dermatitis (L20) in UKB wee performed meta-anlysis with our Icelandic list together with the largest published meta-analysis on AD to date downloaded from the GRASP database (https://grasp.nhlbi.nih.gov/FullResults.aspx), to study this phenotype. For further details, see subsection "Study sample sets for asthma and allergy phenotypes" in Materials and methods.
Recruitment	Asthma in the UK Biobank was defined as ICD10 diagnoses in fields 41202 or 41204, including anyone of J45.0, J45.1, J45.8, J45.9 and J46 and/or self-reported by the non-cancer illness code, self-reported during verbal interview (data-field 20002) with a code for asthma (1111).<br>Icelandic asthma patients over 18 years of age were recruited who attended an asthma clinic or emergency rooom at the National University Hospital of Iceland or the Icelandic Medical Center (Laeknasetrid) during the years 1977 to 2017. Asthma diagnosis was based on a combination of physician's diagnosis and ICD10 diagnosis, including anyone of J45.0, J45.1, J45.8, J45.9 and J46 and/or self-reported by a positive reply to the question: "Has a doctor confirmed your asthma diagnosis". Atopy status determined by skin prick testing and age of onset was available for part of the asthma cohort both in Iceland and UK. Early onset was defined as first diagnosis ≤17 years of age and late onset as first diagnosis >17 years of age.<br>Allergic rhinitis combines doctoral diagnosis of allergic rhinitis from Iceland and a questionnare data from UK Biobank on hayfever or allergic rhinitis (Non-cancer-illness code 1387). As only 42 individuals had the ICD10 code for Atopic dermatitis (L20) in UKB weran meta-anlysis with our Icelandic list (N=8325) derived both from physician's diagnosis and ICD10 code (L20) together with the largest published meta-analysis on AD to date43 downloaded from the GRASP database (https://grasp.nhlbi.nih.gov/FullResults.aspx), to study this phenotype. Other allergy diagnosis were based on ICD10 codes from UKB and either physician's diagnosis or ICD10 codes from Iceland. The allergy phenotypes used were: Nasal polyps (ICD10:J33), chronic sinusitis (ICD10:J32), chronic sinusitis with nasal polyps (combined: ICD10:J32 and J33) and chronic sinusitis without nasal polyps (ICD10:J32 without ICD10:J33). Number of genotyped individuals in each cohort are listed in Supplementary table 12. Icelandic controls were participants from various deCODE genetics programs.
Ethics oversight	All participating individuals who donated blood signed informed consent. The personal identities of participants were encrypted using a third-party system approved and monitored by the Icelandic Data Protection Authority. The study was approved by the National Bioethics Committee in Iceland.

Note that full information on the approval of the study protocol must also be provided in the manuscript.

Flow Cytometry

Plots

Confirm that:

☒ The axis labels state the marker and fluorochrome used (e.g. CD4-FITC).

☒ The axis scales are clearly visible. Include numbers along axes only for bottom left plot of group (a 'group' is an analysis of identical markers).

☒ All plots are contour plots with outliers or pseudocolor plots.

☒ A numerical value for number of cells or percentage (with statistics) is provided.

Methodology

Sample preparation	EBV cells were harvested from culture, counted and diluted to 1x106 cell/ml plated in a 96 well V bottom plate. Cells were then stained with primary antibodies against CD30 (Biolegend 333918) and analyzed for expression of CD30 by FACS. Cryopreserved PBMC were thawed and and plated for culture in RPMI1640 (ThermoFisher 61870-036) supplemented with 10% fetal bovine serum (ThermoFisher 10500-064), 50 units/mL penicillin, 50 µg/mL streptomycin (ThermoFisher 15070-063), 20 mM HEPES

	(ThermoFisher 15630056) and 30 IU/ml IL-2 (R&D Systems AFL202) in a 24 well TCR plate and rested overnight. Cells were then stimulated for 48 hours with anti CD3/CD28 dynabeads (ThermoFisher 11131D). At 48 hours cells were harvested and stained with directly conjugated antibodies (Biolegend 333906, Biolegend 300318, Biolegend 300530, Biolegend 353210, Biolegend 304112) and analyzed for expression of CD30 by FACS. Wilcoxon signed rank test was used to test for significant differences in cell surface expression of CD30.
Instrument	Thermo Fisher Life technologies Attune NxT
Software	FlowJo v10
Cell population abundance	*Describe the abundance of the relevant cell populations within post-sort fractions, providing details on the purity of the samples and how it was determined.*
Gating strategy	Cells were first gated by size with FSC Area/SSC Area and doublets were excluded by gating at FSC Height/FSC Area. Then cells were gated for CD30 expression.

☒ Tick this box to confirm that a figure exemplifying the gating strategy is provided in the Supplementary Information.
