# Supplementary material for: Eighty-eight variants highlight the role of T cell regulation and airway remodeling in asthma pathogenesis
Source: Nat Commun. 2020 Jan 20;11:393. doi: 10.1038/s41467-019-14144-8 (PMC6971247; doi:10.1038/s41467-019-14144-8)
Supplement: Supplementary file 18 — Description of Additional Supplementary Files [file 41467_2019_14144_MOESM18_ESM.pdf]

Title: Supplementary Data 1:
Description: GWS signals associating with asthma in Iceland-UK meta analyses.

Title: Supplementary Data 2:
Description: Known variants (LD r2≥0.2) at previously reported loci

Title: Supplementary Data 3:
Description: Unreported variants (LD r2≥0.2) at previously reported loci

Title: Supplementary Data 4:
Description: Replication of the 36 previously published asthma loci of European-ancestry in Icelandic UK meta-analysis.

Title: Supplementary Data 5:
Description: Novel asthma variants at previously reported loci reported in Ferreira et al. (PMID:29083406) for a combined phenotype of asthma, hay fever and eczema.

Title: Supplementary Data 6:
Description: Whole blood eQTL variants correlated (r2≥0.8) with astma lead variants

Title: Supplementary Data 7:
Description: Coding variants strongly correlated (r2 > 0.8) with novel asthma associated sequence variants

Title: Supplementary Data 8:
Description: eQTL variants correlated with novel astma lead variants found in whole blood, esophagus mucosa, Lung, EBV-transformed lymphocytes and Transformed fibroblasts in the Gtex database.

Title: Supplementary Data 9:
Description: Functional annotations of previously unreported asthma variants

Title: Supplementary Data 10:
Description: Classical HLA alleles correlating with asthma in the Icelandic cohort

Title: Supplementary Data 11:
Description: Effect of genome wide significant Asthma variants on eosinophil count.

Title: Supplementary Data 12:
Description: Effect of the 88 GWAS significant asthma associating variants on asthma subphenotypes and other allergic diseases

Title: Supplementary Data 13:
Description: Genes prioritized with DEPICT pathway analysis

Title: Supplementary Data 14:
Description: Gene sets found enriched in the 88 GWS asthma risk variants using DEPICT pathway analysis
